# Managing Spatial Selections With Contextual Snapshots

**DOI:** 10.1111/cgf.12406

**Published:** 2014-06-20

**Authors:** P Mindek, M E Gröller, S Bruckner

**Affiliations:** 1 Vienna University of TechnologyAustria; 2 VRV is Research CenterAustria; 3 University of BergenNorway

**Keywords:** interaction, visual analytics, spatial selections, annotations, I.3.3 [Computer Graphics]: Picture/Image Generation—Viewing algorithms; I.3.6 [Computer Graphics]: Methodology and Techniques—Interaction techniques

## Abstract

Spatial selections are a ubiquitous concept in visualization. By localizing particular features, they can be analysed and compared in different views. However, the semantics of such selections often depend on specific parameter settings and it can be difficult to reconstruct them without additional information. In this paper, we present the concept of contextual snapshots as an effective means for managing spatial selections in visualized data. The selections are automatically associated with the context in which they have been created. Contextual snapshots can also be used as the basis for interactive integrated and linked views, which enable in-place investigation and comparison of multiple visual representations of data. Our approach is implemented as a flexible toolkit with well-defined interfaces for integration into existing systems. We demonstrate the power and generality of our techniques by applying them to several distinct scenarios such as the visualization of simulation data, the analysis of historical documents and the display of anatomical data.

## 1. Introduction

Visual analysis of large data sets often requires displaying of various subsets of the examined data using different visualization techniques. For this purpose, linked views and integrated views are commonly employed. The investigation of multivariate or otherwise complex data may require a specification of spatial regions which are to be examined individually using integrated or linked views. Creating spatial selections, or brushing, is a widely used method for specifying such regions. Brushing techniques are also often employed to specify a *degree of interest* (DOI) function for focus + context visualization as described by Furnas [Fur86]^1986^. Smooth brushing concerns the specification of a non-binary DOI function, which defines a continuous transition between focus and context data.

Another aspect of visualizing complex data sets is a frequent need for creating annotations in the rendered images. The annotations assign semantics to parts of the image. They are useful for providing additional insight into the data, or for keeping provenance information. The annotations are related to the spatial selections, since they refer to particular spatially bounded image regions. The annotations are also bound to the current parameter settings of the visualization. For instance, if a structure is annotated in a volume data set, the annotation loses its meaning when the transfer function, the viewing angle or other parameters change in such a way that the structure in question is no longer visible. In such cases, it is necessary to keep track of the visualization settings together with the annotations.

We propose a method for managing arbitrary selections in the image space of the visualizations. We define several terms for the purpose of describing the proposed method. A *selection* is a non-binary DOI function in image space. A *visualization snapshot* is a set of parameter values describing the state of the visualization system at a particular point in time. Finally, we introduce the concept of *contextual snapshots*. A contextual snapshot is an entity which holds multiple selections together with a visualization snapshot. The visualization snapshot provides context for the associated selections. By keeping the selections in contextual snapshots, it is possible to recover the states of the visualization system in which the selections have been created. The contextual snapshots allow us to work with multiple selections created in different states of the visualization system.

To demonstrate the proposed concept, we implemented the contextual snapshots in three scenarios, one of which is a volume visualization application. The application displays a multivariate 4D hurricane data set. The user can create selections in image space by using a lasso metaphor. The selected data can be further analysed using linked views. We use this example to introduce the parts of which the contextual snapshots consist, and explain how they work together.

In applications such as 4D data visualization, it is sometimes necessary to select and annotate the visualized data multiple times while the visualization parameters are changing. Contextual snapshots provide a basis for keeping track of these interactions. In current systems, selections made in image space have to be processed before the image changes. Otherwise the selections will become invalid with the new parameter settings, which we refer to as context. Contextual snapshots record the selection together with the context, so that it can be processed even after the context has changed.

This paper is based on our previous work [MBG13][Bibr b20]. It contains an extended description of the concept of contextual snapshots for managing multiple spatial selections in different steps during the visualization session. We extended the description of our implementation of the contextual snapshots. We include details on implementation efforts needed to use contextual snapshots in an existing visualization system. The library can be downloaded from our website [csl]^2014^. There is also a detailed tutorial which explains on a simple example how to integrate contextual snapshots into an existing application. We extended the implementation with new, customizable anchors, which serve as abstract previews of the contextual snapshots. We added a possibility to use custom matching functions which define when individual contextual snapshots are active. Finally, we provide a new use case, which shows contextual snapshots applied to the visualization of 3D geometry data.

## 2. Related Work

We propose a method for managing spatial selections in image space. There are various scenarios where multiple selections are made in order to achieve a certain goal. The goal might be to select subsets or features of a data set. Furnas [Fur86]^1986^ presents DOI functions for the specification of focus data. Doleisch and Hauser [DH02]^2002^ use a DOI function obtained by smooth brushing to modify the visualization mapping in 3D flow visualization. Doleisch *et al*. [DGH03]^2003^ present a framework for the specification of data features visualized in several linked views. Ulinski *et al*. [UZW*07]^2007^ propose two-handed methods for creating selections in volume rendering. Unger *et al*. [UMDS08]^2008^ use smooth brushing in the visualization of statistical characteristics for subsets of large data sets. Various methods for increasing the usefulness of 3D scatterplots incorporating brushing have been developed [KSH04]^2004^, [PKH04]^2004^. Streit *et al*. [SSL*12]^2012^ propose a model-driven design process for exploring multiple linked data sets. Yu *et al*. [YEII12]^2012^ discuss methods for selecting data in large 3D point clouds by screen–space interaction. In visualization applications which employ brushing or similar techniques, the user interaction is typically limited to the common context. Contextual snapshots remove this limitation by providing means for keeping the context for each individual interaction instance.

Gerl *et al*. [GRIG12]^2012^ incorporate brushing on renderings of data attributes for the specification of semantics in volume visualization. Guo *et al*. [GMY11]^2011^ introduce a sketch-based interface for direct volume rendering which replaces the traditional way of transfer function design. Wei *et al*. [WWYM10]^2010^ propose a sketch-based interface for an interactive 3D vector field exploration. The concept of contextual snapshots is designed in such a way that the spatial selections could be employed to handle the just mentioned types of user interaction. Contextual snapshots increase the scalability of such interaction methods. They allow the system to manage multiple interaction instances simultaneously, while each instance can be meaningful in a different context.

In addition to the concept of contextual snapshots, we propose a method for combining them with various views of the visualized data. The integration and linking of multiple views has been extensively explored [Bal10]^2010^, [Tor04]^2004^. Bier *et al*. [BSP*93]^1993^ propose a see-through interface as a natural way of displaying additional data. Balabanian *et al*. [BVMG08]^2008^ introduce a framework for the specification of visualization parameters for time-varying data. Rungta *et al*. [RSD*13]^2013^ present ManyVis—a framework for easy integration of existing applications to create custom visualization tools. Santos *et al*. [SLA*09]^2009^ propose VisMashup, a framework for simplifying the creation of custom visualization applications. The authors of VisMashup combine various visualization pipelines to create a new visualization application. In our work, we aim at extending existing visualization pipelines with interaction possibilities.

Contextual snapshots can also be used for preserving user-created provenance information for a visualization. Bavoil *et al*. [BCC*05]^2005^ propose VisTrails. It is a system for creating and maintaining visualization pipelines with the possibility to execute them and to record their provenance information. Our method differs form VisTrails in that the user can create spatial selections of the explored data in a specific context and annotate them to store the visualization provenance information. This provides a strong link between the provenance information and the underlying data. Heer *et al*. [HMSA08]^2008^ present a design space analysis of history keeping systems. Kreuseler *et al*. [KNS04]^2004^ propose an approach to include a history mechanism into a visual data mining framework. Groth and Streefkerk [GS06]^2006^ present a method for capturing the history of the knowledge discovery process using a visualization system with an ability to create annotations for provenance information. Ellkvist *et al*. [EKF*09]^2009^ discuss an architecture for provenance inter-operability between multiple sources. In contrast to these systems, contextual snapshots provide means to insert annotations to particular spatial data at a particular stage of the visualization session. As the annotations are automatically linked with the current context, they store provenance information besides their actual content.

## 3. Overview of Contextual Snapshots

Many visualization systems use brushing, selections and linked views to provide means for the exploration of complex data sets. There are various tools for specifying the selections and they usually serve only one specific purpose. The idea of contextual snapshots is to harness a single mechanism of 2D spatial selections for different tasks, such as data selection and manipulation, data annotation or specification of DOI functions. In contextual snapshots, this is achieved by the following concepts: multiple selections can be stored and each of them can be created in a different context (i.e. parameter settings of the visualization system); an algorithm of transforming the user input to the DOI functions of the selections is interchangeable; each selection can be linked with a number of additional views which we refer to as *embedded visualizations*. It is possible to display, that is, embed them, directly in the visualization image. Embedded visualizations are interactive and they can display arbitrary graphical user interface elements or visualize data specified by a corresponding selection.

In the example application, the selections can specify a spatial region in image space of a volume visualization. An embedded view which displays the histogram of the volume data in the specified region is linked with each selection. Contextual snapshots calculate a histogram of the selected voxels and provide it to the embedded visualization. How these data are used depends on the implementation of individual embedded visualizations. Another embedded visualization is a simple text field. It does not display the selected data region, but it allows users to type in arbitrary text-based annotations. Such an embedded visualization is linked with every selection, thus providing a possibility to annotate selected data subsets.

### 3.1. Concept of contextual snapshots

A visualization system may apply various parameters to modify the visual mapping. With respect to contextual snapshots, the values of a chosen subset of said parameters, the *visualization snapshot*, define a state of the visualization system. For any state of the system, a user can create several selections in the rendered images. A contextual snapshot stores a visualization snapshot together with all selections created when the state of the visualization system corresponded to this visualization snapshot.

In the hurricane visualization application, we use the position and the orientation of the virtual 3D camera as the parameters stored within the visualization snapshot. Therefore, each image–space selection is bound to one 3D camera view. The selection is valid only if the volume is rendered using this camera view. Other parameters, such as current timestep, are not stored in the visualization snapshots. Changing these parameters does not make the selections invalid. This is application-specific and in the given case, it allows users to explore how the hurricane data change over time in specified spatial regions. When contextual snapshots are integrated with a visualization system, the system integrator has to choose the set of parameters which appropriately define the context for the selections.

In this work, a selection is a function 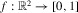 which specifies a DOI for each pixel. The contextual snapshots do not assume any particular definition of this function. Therefore, arbitrary types of image–space selections can be used. This is demonstrated in Sections 5 and 6.

A contextual snapshot 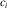 is defined as:

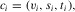
(1)


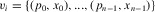
 is a visualization snapshot containing *n* parameters of the visualization system, describing its state at one instant. The visualization snapshot consists of *n* pairs 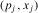, where 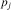 is a parameter name and 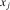 is its value; 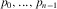 are the same for all contextual snapshots, while the parameter values 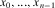 are specific for each visualization snapshot; 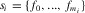 is the set of all selections created at the state, or context, described by 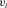. 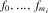 are the user-defined, real-valued selections as described. Finally, 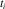 is a thumbnail image of the visualization in the state described by 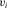.

The idea of contextual snapshots is based on the fact that the semantics of the user-made selections depend on what is currently displayed. When the user selects a particular feature in the image, the selection is meaningful only until the way how the feature is displayed changes. Therefore, we extract a visualization snapshot every time a new selection is created. The visualization snapshot is linked to the selection to create a contextual snapshot. The contextual snapshot then provides a reproducible spatial selection related to what was displayed when the selection was made. It stores the appropriate visualization context in the form of the values of the visualization-system parameters. All selections with the same visualization snapshot are stored together within one contextual snapshot.

The strength of contextual snapshots is that they can maintain multiple selections created in different states of the visualization system. The information stored within a contextual snapshot can be used to restore the given state, so that the selections can be displayed and actively used. By restoring the state of the visualization system according to the individual contextual snapshots, it is possible to browse all selections created within a visualization session.

**Figure 1:**
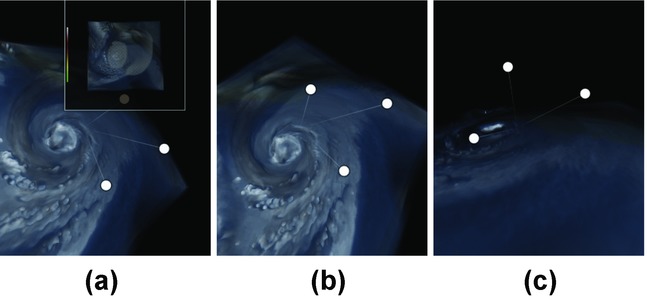
Interactive anchors (white circles) representing individual contextual snapshots. For better 3D orientation, the anchors are connected with the coordinate origin by a thin line; (a) shows how the thumbnail of an anchor can be displayed; (b) and (c) show the anchors from different camera views.

Contextual snapshots are represented by icons which we refer to as anchors. The anchors are embedded in the original visualization as interactive graphical elements. They constitute abstract previews of the corresponding contextual snapshots. For instance, in the hurricane visualization application, the anchors are positioned in 3D space to represent the camera positions when the respective contextual snapshots have been recorded. An anchor can also display a thumbnail of how the visualization looked like when the respective contextual snapshots have been created. The anchors are interactive and they are used to restore the visualization-system state to the respective contextual snapshot. They serve as a user interface for browsing through the contextual snapshots created during the visualization session. Figure 1 shows the graphical representation of anchors.

The visualization-system parameters used as context for the selections vary in different applications. The graphical representation of anchors can be customized to convey the information stored in the contextual snapshot, as demonstrated in Section 6. The default implementation uses the viewing–transformation matrix of the camera to position the anchors in 3D space. In this case, the position of the anchor conveys the position of the camera at the time when the contextual snapshot has been recorded. This is only suitable for visualizations using a 3D camera. For other use cases, we allow programmers to set an arbitrary screen–space position for each anchor. This way, the anchors can be positioned with respect to a feature of the visualization to potentially reveal the semantics or content of the contextual snapshot. This is demonstrated in the use case described in Section 5.

### 3.2. Embedded visualizations as linked views

To broaden the possibilities of using context-aware selections, we provide a method for linking interactive embedded visualizations for each selection. The additional visualizations can show different aspects of the selected data, or they can display comparisons of various selected areas. To demonstrate different ways how the selections can be used, we implemented the following embedded visualizations for the hurricane visualization application: a histogram of selected data values, a text-based annotation widget and a variable picker.

**Figure 2:**
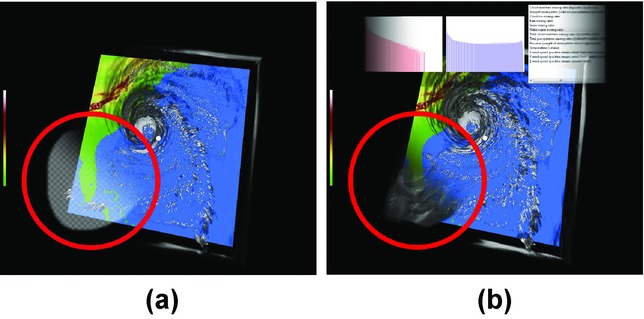
(a) A selection (marked with the red circle). (b) An integrated view of two variables using the selection after its activation. Histograms are shown for both variables as embedded visualizations. The third embedded visualization is a variable picker—a list of the data variables, where the user can choose which one is displayed.

The embedded visualization displaying the histogram of selected data values can be linked to multiple selections at once. It shows histograms for individual selections in overlays so that they can be easily compared. A separate text-based annotation widget is linked to each selection. It provides the user with the possibility to type in a short description of the selected data subset. The variable picker is a graphical user interface element which displays a list of all variables present in the data set. The chosen variable is displayed in those parts of the image, where the selection has been created. Figure 2 shows how the picked variable is integrated with the rest of the visualization by a smooth transition. The smooth transition is due to the non-binary DOI function of the selection.

In our method, each image–space selection can be linked with multiple embedded visualizations. Each embedded visualization has access to the data subsets specified by the selections to which they are linked. A single embedded visualization can be used to display and compare aspects of different subsets of the explored data by simply linking it with multiple spatial selections.

For the purpose of displaying the embedded visualizations, we propose a mechanism for activating individual selections. One or multiple selections can be activated by the user at once. In this case, only those embedded visualizations are displayed which are linked with every activated selection. The rationale for this mechanism is that the embedded visualizations can show different aspects of the data specified by multiple selections at once. This way, the selections and their embedded visualizations can be used to compare several data subsets.

A sketch-based interface is used for activating selections. The user activates selections by painting a stroke in image space. The selections which are crossed by the stroke are activated and subsequently their linked embedded visualizations are displayed. The embedded visualizations are grouped together in a sliding bar, which is displayed either at the top, the bottom, the left or the right side of the visualization image. The position of the sliding bar is determined by the direction at the end of the stroke which was used to activate the selections. The interaction method of using a stroke was chosen so that an arbitrary subset of the selections can be activated, which might be difficult with various standard selection mechanisms.

The sliding bar is capable of showing several embedded visualizations at once. In case there are more embedded visualizations for the activated selections than actually fit on the screen, the sliding bar enables scrolling of its content. The scrolling is executed by an animated transition, so that the users have a visual feedback on the direction of the scrolling. The sliding bar fades to the original visualization on both sides for better integration. A gradual blurring filter was used on both sides of the sliding bar, so that the attention of the users is guided to the embedded visualizations currently shown in the middle of the bar.

Displaying several embedded visualizations at once provides users with an overview of the additional data depicted for the activated selections. However, to facilitate interaction it might sometimes be necessary to enlarge individual embedded visualizations. For this purpose, the embedded visualization displayed in the middle of the sliding bar can be switched to a so-called maximized view. If the view is maximized, the embedded visualization is displayed on the whole screen rather than just in the sliding bar.

## 4. Contextual Snapshot Architecture

Contextual snapshots are meant to be used in existing visualization systems. We have implemented contextual snapshots as a library which can be integrated with an underlying visualization system on the source-code level. We call it Contextual Snapshot Library (CSL). The CSL is responsible for rendering the anchors, the selections and the embedded visualizations into the original visualization image. Additionally, it provides an interface for the data transfer between the selections and the embedded visualizations. It also handles user input so that the anchors and embedded visualizations are interactive. Contextual snapshots integrate the visualization and the graphical elements for interactive data exploration and annotation. This approach is particularly well suited for the rapidly growing area of mobile devices such as tablets where the display also serves as the input device.

**Figure 3:**
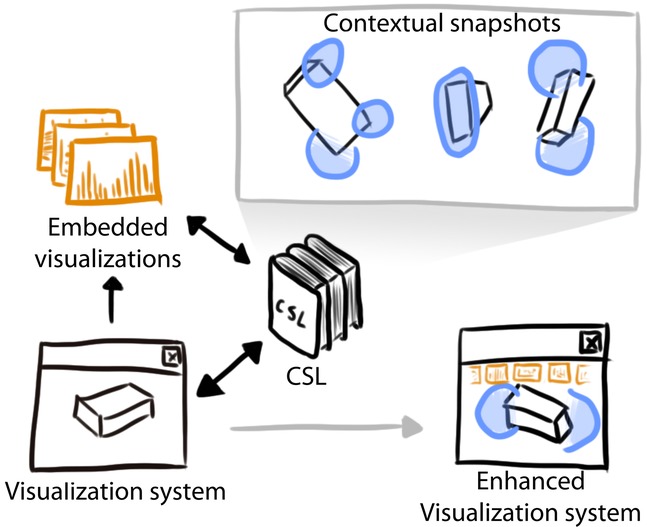
Overview of the system. The black arrows represent the data flow between the visualization system, the embedded visualizations and the Contextual Snapshot Library (CSL). The grey arrow denotes the transition from the original rendering to the rendering with the enhanced visualization.

Figure 3 shows the data flow between the visualization system, the embedded visualizations and the CSL. The visualization system gathers user input and transmits it to the CSL. The CSL stores contextual snapshots generated from the user input. Additionally, it renders the selections, the anchors and the embedded visualizations into the original visualization image. The result is an enhanced visualization system.

The CSL renders all of the graphical elements (anchors, selections, embedded visualizations, as illustrated in Figures 1 and 2) into the original visualization and provides the result as a texture. The underlying visualization system can be modified to display this texture so that the graphical elements of the contextual snapshots are visible.

**Figure 4:**
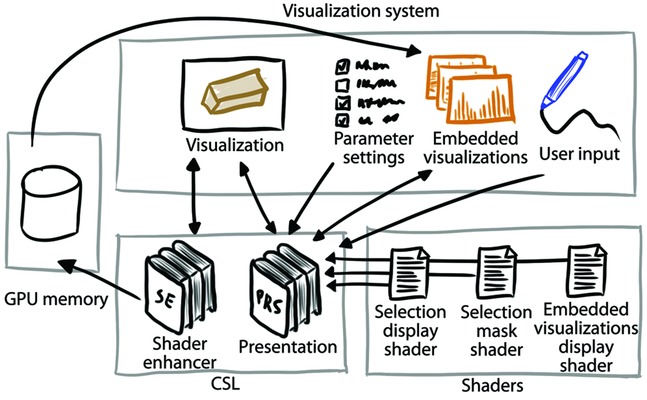
Architecture of the visualization system integrating the CSL. The arrows denote the data flow.

Figure 4 shows the overall architecture of an existing visualization system using the CSL. The library itself is split into two parts. The part responsible for managing the contextual snapshots, interaction and rendering of the graphical elements, is called Presentation (PRS). To exploit the capabilities of modern GPUs, the PRS uses several shaders to render all the graphical elements. The selection mask shader transforms user input into the DOI function of the selection. The selection display shader renders the selection on the screen. The embedded visualizations display shader renders the sliding bar. Each of these shaders can be exchanged to modify how selections are treated.

The functionality of the PRS can be extended by the Shader Enhancer (SE). The SE is an auxiliary tool for the data transfer between individual modules of the visualization system. It stores data specific to individual selections of the contextual snapshots in the GPU memory so that it can be used in different visualization pipelines of the system. The SE simplifies the utilization of the selections in the visualization system by providing access to all data subsets specified through the corresponding DOI functions. The motivation of storing the data in the GPU memory is that GPU implementations of visualization algorithms can access the data without having to transfer them to CPU memory.

The CSL is implemented in C++, using the Qt library. It uses OpenGL for rendering of the graphical elements and for the data exchange with the visualization system via textures. The specifications of contextual snapshots can be stored on the hard drive in XML format. The selection masks and the thumbnails are stored as PNG files. The CSL also provides functionality to load this information and recreate all the contextual snapshots for the currently visualized data.

### 4.1. Contextual snapshots

When the parameters of the visualization system are set so that their values match the values stored in visualization snapshot 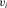 (as described in Section 3.1), the contextual snapshot 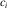 is activated. The default implementation requires that each pair of corresponding values are equal in order to activate the respective contextual snapshot. However, it is possible to provide custom matching functions for each parameter data-type or even for individual parameters. The custom implementation of the matching functions can treat parameter values, whose difference lies in a certain range, as a match. This way, the contextual snapshot 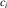 is activated even if the parameter values set in the visualization system are not exactly equal to those stored in 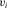, but the values of the corresponding parameters are similar enough.

Another possibility to activate contextual snapshots is by using anchors. The visualization system can request the CSL to select an anchor which is displayed at a certain position in image space (e.g. on mouse click). The contextual snapshot represented by the selected anchor becomes active. The state of the visualization system is changed so that it corresponds to the active contextual snapshot. All selections belonging to this contextual snapshot can now be displayed.

The activation of the contextual snapshot is accompanied by an animated transition from the values of the parameters it stores to the current values in the present state of the visualization system. For individual parameters, different transition functions can be used in order to achieve an appropriate interpolation for a specific data type (e.g. Slerp for rotation matrices). This enables smooth transitions between system states while switching between them. In the hurricane visualization application, the state of the visualization system is defined by the camera position and orientation. Therefore, the activation of a contextual snapshot causes the visualization system to smoothly change the 3D camera to the view with which the contextual snapshot was recorded.

Internally, parameter values are represented by Qt's QVariant class, so that any parameter type can be used. However, transition and matching functions have to be implemented for every type. The CSL provides implementation of these functions for several common types (e.g. integer and real numbers, transformation matrices, 2D and 3D vectors). These can be modified to meet specific requirements from individual applications. New transition and matching functions can be added by the system integrator to extend the CSL with new parameter types.

### 4.2. Selections

Selections are created by calling functions of the CSL's API. If the contextual snapshot 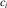 is active, the newly created selection is automatically assigned to it, that is, it is added to 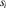 (Equation (1)). Otherwise a new contextual snapshot is created from the current values of the visualization-system parameters and the selection is assigned to it. This mechanism enables multiple selections created in the same context to be assigned to a single contextual snapshot.

The process of creating a selection consists of three steps: recording of the user input, transforming the input to a DOI function in image space, displaying the DOI function on screen to represent the selection. In our approach, we made a clear separation between these steps. Each of them is implemented as a stand alone shader program with clearly defined input and output. Because of this separation, it is possible to customize the process of creating selections for various applications. Examples are selections using the lasso metaphor or rectangular selections. In both cases, the only component of the CSL that is exchanged is the shader realizing the transformation of the user input to the DOI function.

If a user interacts with the visualization system to create an image–space selection, a series of points in the image space is recorded. We refer to this series as a selection stroke. The selection stroke can be created by mouse, graphics tablet or a similar input device. The CSL transforms the selection stroke to a grey-scale mask representing the non-binary DOI function. Pixel luminosity encodes the DOI in the respective point. This selection mask is generated by a selection mask shader. The input of the shader program is the selection stroke encoded in a 1D texture. The output of the shader program is the selection mask.

**Figure 5:**
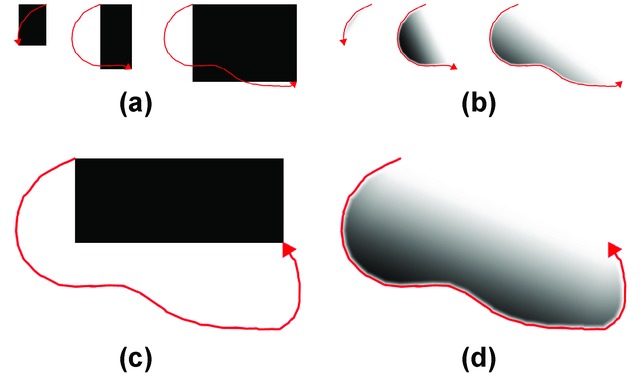
Two different selection mask shaders generate selection masks (black means fully selected, white means not selected at all) for the same selection stroke (in red): (a) and (b) illustrate sequences of creating the stroke, while the selection mask is continuously generated at every step; (c) and (d) illustrate the final selection masks for the given stroke. The gradual change in the level of selection enables smooth brushing.

Depending on the shader program used to generate the selection masks, the selections may enable the visualization system to realize smooth brushing. We provide several different shader programs for generating the selection masks. Figure 5 shows how the selection masks generated by two different shaders look like for the same selection stroke. The shader illustrated in Figure 5(c) creates a simple rectangle based on the first and the last point of the stroke. This is a common way to create a rectangular selection. The shader shown in Figure 5(d) uses the so-called lasso metaphor. The stroke defines a closed polygon whose interior is filled. We enhance the lasso metaphor to account for selection uncertainty by introducing a smoothing of the edges. The smoothing between the first and the last point of the stroke is stronger if these two points are farther away. The other edges are smoothed by a constant factor. This enables users to control the amount of smoothing as well as its spatial location. The motivation for such an approach is that if a user does not fully enclose the selected region, the selection status of the area between the beginning and the end of the stroke remains uncertain.

**Figure 6:**
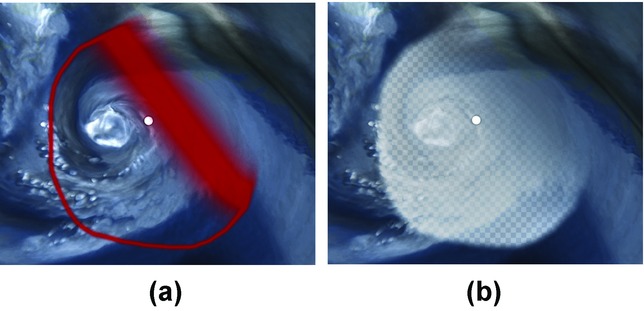
Two different selection display shaders. Both shaders are able to show the fuzziness of the selection.

The selections are displayed on the screen using the selection display shader. The way how the selections are displayed is important for specific applications. Figure6 shows two different choices how the selections can be displayed. In Figure6(a), an example of outline drawing is shown. The red colour marks borders between selected and unselected areas. The thickness of the border denotes the uncertainty of the selection in that particular area. In Figure 6(b), the selection is displayed using an overlay texture. This method shows the selected area in a clear way, but it also partially occludes displayed data underneath.

### 4.3. Data transfer

The selection masks are stored in the GPU memory as a texture array so that the visualization system can access them at all times and employ them in the visual mapping. We used this ability in the hurricane visualization application to create a smooth transition between the two visualized variables.

The SE can be used to further increase possibilities of the selections. It allows the visualization algorithm to extract processed data samples to the GPU memory. The activated selections specify the DOI, which is used to automatically weight every extracted data sample. The extracted data can then be accessed in the embedded visualizations.

Currently, we provide an implementation of the SE for GLSL shaders. The SE inserts GLSL code for the extraction of data samples and their weighting by the selections' DOI functions in the visualization shader before it is compiled. The inserted code uses atomic operations and the GL_EXT_shader_image_load_store extension to output desired data. After the extraction, the data are available to the embedded visualizations as a texture stored in the GPU memory. It is possible to extend the SE for other languages as well.

## 5. Application Example: Historical Document Analysis

In addition to the example application introduced in Section 1, we present two more use cases of contextual snapshots. In the first one, we take a simple book reader application. A page spread consisting of two pages of a manuscript is displayed. A bar showing the current page within the manuscript is located below the pages. For a better user experience, a simple page turning animation is realized whenever the current page changes.

We combine our method with the described book reader application in order to add functionality enabling the users to employ it as an advanced manuscript analysis tool. We used the CSL to implement spatial selections on the displayed pages. The CSL automatically binds every new selection to the current page, so many selections on different pages can be created.

In this example, we demonstrate how the selections can be incorporated into an existing system, how anchors can be positioned on the screen to serve as bookmarks for individual selections, and how the data specified by the selections can be displayed in the embedded visualizations. This example also demonstrates the possibility to compare data from multiple selections in the same embedded visualization. The embedded visualization can further contain various interactive elements, such as JavaScript-enabled web pages.

### 5.1. Manuscript visualization

The data set taken in this example consists of 723 high-resolution photographs of pages of the Venetus A, a 10th century (AD) manuscript of the Iliad catalogued as Marcianus Graecus Z. 454, now 822. In addition to natural light photographs, some of the pages were recorded using UV photography as well. The UV light photographs were taken in order to reveal some details of the manuscript which were hardly visible with natural light. Together with the photographs, the transcript of the Iliad in ancient Greek was available as well.

For demonstration purposes, we manually pre-processed the acquired data. Some of the UV photographs were registered with the natural light photographs so that they could be easily used in the application. Additionally, appropriate passages of the transcript were matched with some of the photographed pages.

The described book reader application is only capable of displaying the natural light photographs. The book reader shows an icon for those pages where the UV data are available. The goal in this example is to use the CSL to display parts of the UV photographs on selected regions of interest. The regions of interest are rectangular areas specified for the pages with UV data available.

### 5.2. Manuscript visualization enhancement

**Figure 7:**
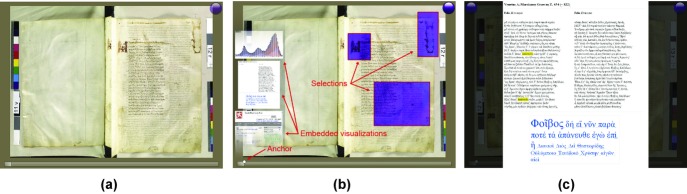
(a) The book reader application displaying the Venetus A manuscript. The icon (violet circle) on the top right corner indicates the availability of UV light data for this particular page. (b) The book reader application enhanced with the CSL to show additional data. An anchor of a contextual snapshot, selections and embedded visualizations are displayed. (c) The transcript of the displayed pages with the tag cloud in the maximized view. A word selected in the tag cloud is highlighted in yellow.

Figure 7(a) shows the book reading application without the enhancements. Figure 7(b) depicts the application enhanced by using the CSL. The contextual snapshots were used to manage the selections. The selections are employed to show parts of the UV photograph of the page. The transcript of the page as well as colour histograms of the selected parts of the natural light and UV photographs are displayed on the left side of the image. These additional visualizations are linked with the activated selections. The activated selections are concurrently used to display parts of the registered UV photograph of the selected page. Any of the additional visualizations can be maximized, as shown in Figure 7(c).

The only parameter with an impact on the manuscript visualization is the index of the current page. This parameter determines which pages of the manuscript are displayed. As the turning of the pages is animated, we allow the current page index to be a real number. The fractional part of the index is used for the animation of the page turning.

Parts of the displayed pages can be selected. The selection is meaningful only for the page in which it was created. The current page index constitutes the visualization snapshot for this application, because it alone fully describes the context for the selections. The current page index is visualized by the book reader as a slider displayed below the pages. The anchor of each contextual snapshot is placed on the slider according to the current page index. The anchor therefore visually represents the position of the displayed page spread and can be used as a bookmark.

The rectangular selections support the visual analysis of interesting parts of the photographs. By clicking the UV icon, displaying of the UV light photographs in the selections can be enabled or disabled. As the UV and natural light photographs are co-registered, the selections create a comprehensible integrated view.

We have implemented three web views which give web pages as embedded visualizations. With the web pages, we demonstrate that the embedded visualizations managed by the CSL are interactive and that they can contain arbitrary content. The first web view contains colour histograms from the selections. As multiple selections can be activated, we employed the JavaScript library D3 [BOH11]^2011^ capable of displaying multiple histograms at once. For each selection, a colour histogram is displayed. For the pages where the UV data are available, the histograms from the UV photographs are displayed as well. As all of the histograms are given in one view, they can be easily compared.

The second web view shows the Greek transcript of the displayed pages. Contextual information of the selections, that is, the current page index, is used to load the appropriate pages from the transcript. A tag cloud of the most frequent words generated by the JavaScript is displayed below the text.

The third web view contains a web page of the Perseus Word Study Tool [Mah01]^2001^. This web application provides an English translation of a specified Greek word, as well as further information. We have connected this view with the Greek transcript of the displayed pages. The user can double-click on any word in the transcript to automatically display its definition with the Perseus Word Study Tool.

The application of the CSL in the book reader example demonstrates various ways how the contextual snapshots integrate different views of the visualized data. This use case contains several types of annotations which can be helpful in analysing the historical manuscript. It shows that the integration of vastly differing visualization techniques including online content and GPU-based rendering is easily possible with our approach.

## 6. Application Example: Heart Visualization

In order to illustrate the possibilities of the CSL and the contextual snapshots in general, we apply it to extend an existing visualization system. We choose *VolumeShop* [BG05]^2005^, a powerful visualization system with a modular architecture. We extend a VolumeShop plug-in for rendering 3D meshes with contextual snapshots to create an application where it is possible to easily add annotations for individual parts of the geometric model.

In this example, we demonstrate how user input can be transformed to different types of selections and how anchors can be customized to provide abstract previews of stored contextual snapshots. Additionally, we show how the stored selections can be used in the visualization mapping. Finally, we demonstrate how the embedded visualizations can be employed as informative annotations of the selected data.

### 6.1. Heart visualization

**Figure 8:**
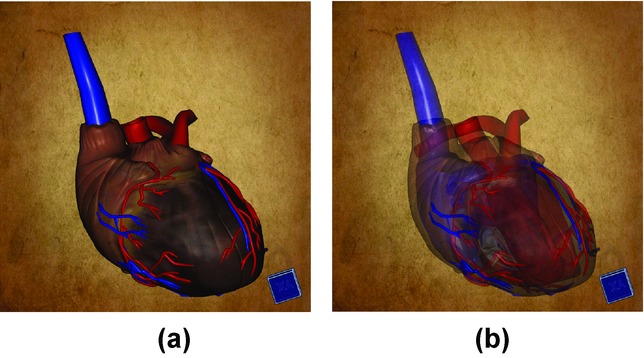
(a) A 3D model of a human heart displayed in VolumeShop. (b) The model is displayed with a lower opacity to reveal the internal structures.

In our example, a VolumeShop application displays a 3D model of a human heart. The model is composed of 32 parts representing individual anatomical structures. It is possible to freely rotate, zoom and pan the model to look at it from different viewpoints. The rendering algorithm also allows us to specify the opacity of the displayed model parts to reveal occluded structures. Figure 8 shows how the heart model is displayed in VolumeShop.

### 6.2. Anatomical annotations

In this example, our goal is to allow domain experts to annotate individual parts of the displayed 3D model. The application can be presented to non-expert users who can interactively explore the model and learn the names of the individual constituting parts.

The CSL extends the interaction possibilities of the VolumeShop plug-in. The basis for the annotations are spatial selections created in the image-space of the 3D model visualization. The context of the selections consists of the camera viewpoint and the opacity value used for the rendering. If either of these parameters changes, the selections might not encompass the desired structures any more, and therefore they disappear. Each selection is assigned to a contextual snapshot, which stores the respective camera viewpoint and the opacity value.

**Figure 9:**
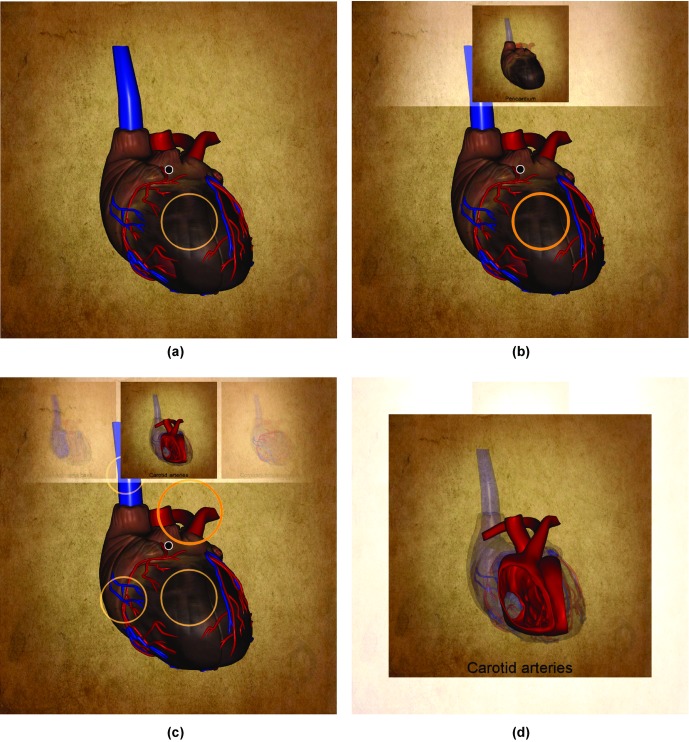
(a) The user creates a circular selection and provides a caption for it. (b) When the selection is activated, an embedded visualization of the selected object in the context of the original model is shown with the specified caption at the bottom. (c) Multiple selections in the same context (viewpoint and opacity value) can be created. All of their embedded visualizations are shown at once in the sliding bar. The one in the middle is highlighted, as well as its associated selection (bold yellow circle). (d) The highlighted embedded visualization is maximized for a better view. The embedded visualizations can be browsed in the sliding bar or in the maximized view.

For each created selection, a list of all objects visible within the selection is composed. The users indicate which of these objects did they intend to highlight with the selection. They can also specify a caption which will be paired with this selection. Afterwards, a rendering is created where all structures of the 3D model are semi-transparent. It is overlaid with the user-selected objects rendered in full opacity. The specified caption is displayed underneath. This rendering is then depicted in an embedded visualization assigned to the selection. This way it is possible to annotate individual objects or groups of objects, which are then given in the context of the original 3D model. This process is illustrated in Figure 9.

We implemented the object selection through the stencil buffer and occlusion queries. After creating a selection, each object is displayed separately with the selection rendered in the stencil buffer. Using occlusion queries, we determine whether any of the pixels were rendered to the framebuffer. If so, the given object intersects the selection and it is listed as one of the selected items. The user can decide which of these objects were meant to be selected, since the selection might also intersect objects which are not of interest. This demonstrates how individual selections can be used within the host system to implement new functionality.

### 6.3. Anchors

The anchors are placed in the 3D space so that they convey the viewpoints associated with the respective contextual snapshots. The context also contains an opacity value. In case only the opacity value changes but not the viewpoint, several anchors representing different contextual snapshots can be placed at the same 3D position.

**Figure 10:**
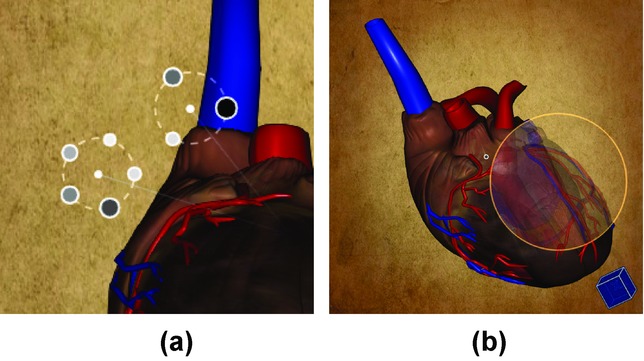
Custom usage of graphical elements of the CSL in the heart example: (a) Two groups of otherwise overlying anchors are displayed in a circular layout around the groups' centres. (b) Hovering the mouse pointer over an anchor shows the selections from the respective contextual snapshots in an integrated view.

To alleviate the occlusion problem, we offer the possibility to customize how the anchors are rendered. If two or more anchors are placed at 3D positions which are projected to similar image–space positions, they are grouped together and are displayed on a circle around the centre of the group, as shown in Figure 10(a). This way, occlusion of anchors is avoided and it is possible to interact with all of them. For detecting anchors on similar positions, we use the DBSCAN clustering algorithm [EKSX96].

Additionally, we enhanced the customized rendering of the anchors by conveying the opacity value stored within the respective contextual snapshots. The difference between the stored opacity value and the opacity value of the current system state is mapped to the anchor's size and colour. Black means the anchor was created with the same opacity value as given in the current system's state. White means the current opacity value is largely different from the one stored within the contextual snapshot. The darker colours of the anchors are also emphasized by larger sizes of the anchors.

### 6.4. Selections

A default behaviour of the CSL is to show a thumbnail of the visualization if the user hovers the mouse pointer over an anchor. This functionality enables users to quickly browse through recorded contextual snapshots and choose relevant ones for the data exploration. We extended this functionality to ease the exploration of the contextual snapshots. If the current viewport of the visualization system is very close to one stored within a contextual snapshot, hovering the mouse pointer over its anchor will not simply show the thumbnail. The selections of the contextual snapshot are rendered, and the selected areas are overlaid with the rendering of the model with the opacity value stored in the contextual snapshot. This creates a meaningful integrated view of the current rendering and the rendering of the contextual snapshot, because both viewpoints are very similar or equal. This functionality is illustrated in Figure 10(b).

**Figure 11:**
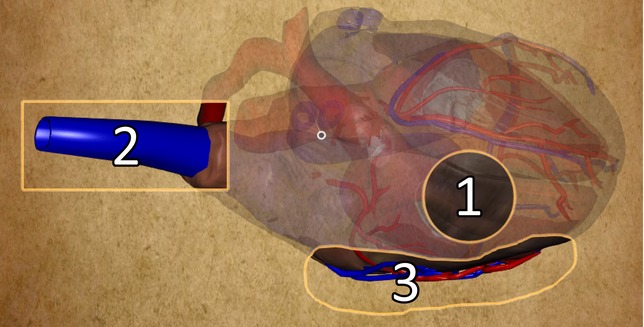
Different types of selections available in the heart application: circular (1), rectangular (2), free-hand lasso (3) selections.

As the application in this example deals with annotating anatomical structures, the circular selection might not always be suitable. Therefore, we implemented three different types of spatial selections: circular, rectangular and free-hand lasso ones. The user can choose the type of selection before its creation. This functionality also demonstrates the extensibility of the CSL. Different types of selections can be specified using simple external shader programs, which can be easily interchanged at run-time. All three types of selections are illustrated in Figure 11.

## 7. Integrating the CSL with Existing Visualization Systems

Our implementation of the CSL uses the Qt library. It is necessary to link Qt together with the CSL to the host system. In our examples, the Qt library was already part of the host visualization systems, therefore there were no additional dependencies.

The CSL provides functions for creating and accessing the selections, activating them and adding embedded visualizations. However, there are no functionality for handling user input. It is necessary to implement handling of keyboard and mouse events, and call-relevant functions from the CSL in the respective event handlers.

It is necessary to modify the rendering pipeline of the visualization system in such a way that the output is rendered to a texture, which is provided to the CSL as input. The CSL then renders all visual elements (anchors, selections and embedded visualizations) with the input texture as background into an output texture. The output texture is sent back to the visualization system. The rendering pipeline of the visualization system should be extended by displaying the output texture of the CSL on the screen instead of its original output. This is usually fairly easy to achieve by taking a framebuffer object as a rendering target. Afterwards, a quad is rendered which covers the whole screen and which is textured with the output texture of the CSL.

In the heart visualization example, the rendering of the anchors is customized. This is done by subclassing a CSL class responsible for the rendering of the visual elements, and overriding the function for rendering of the anchors. The subclass also implements handlers which react to events from the CSL, such as requests to update in case visual elements of the CSL need to be repainted. If the host visualization system uses Qt, an alternative way for this would be to use Qt's signal/slot mechanism.

## 8. Discussion

The goal of our work is to introduce a general concept for handling spatial selections created in changing contexts during a visualization session. Instead of realizing a new standalone system, we implemented this concept as a flexible toolkit, that is, the CSL. The presented examples demonstrate that the CSL is ready to be integrated with different existing visualization systems. Section 7 describes the implementation efforts needed for the integration of the CSL in the provided examples.

In Sections 5 and 6, we give examples how the concept of contextual snapshots can be employed. State-of-the-art visualization systems usually treat selections in such a way that it is necessary to use several linked views to work with multiple selections simultaneously. To employ selections as interactive annotations, each selection would have to be assigned a separate view, possibly in a separate window. Contextual snapshots allow us to realize multiple selections in the same view while the changes of the visualization are automatically tracked. Our method does not provide a guidance for finding appropriate views or means for selecting the data automatically. It extends the common possibilities of data selections to act as data annotations, to convey and to communicate users' findings. In this way, the contextual snapshots support users in the data exploration process.

By employing the CSL to render the selections, the anchors and the embedded visualizations, the performance of the rendering dropped from 60 FPS to 30 FPS in the historical document analysis example. The performance drop of the whole system mainly depends on the temporal requirements of the embedded visualizations. This aspect can be improved in the future by parallelizing the rendering of individual embedded visualizations. In the heart visualization example, there is no significant performance drop after integrating the CSL, since the embedded visualizations only show static images.

We encourage the usage of the CSL, since contextual snapshots can be beneficial for a wide variety of applications. Therefore, we made the CSL available [csl]^2014^. There a detailed tutorial explains with a simple application case how to ingrate the CSL into existing visualization systems.

## 9. Conclusion

In this work, we proposed a method for managing image–space selections which can be used for various tasks, such as highlighting of interesting regions in visualizations, displaying additional views for selected data or comparing different spatial regions. We demonstrated the utility of the method by applying it to three distinct use cases, namely analysis of a historical manuscript, analysis of multivariate weather simulation data and annotation of the geometrical model of a human heart.

Our method is meant to be applied to visualization systems where the state changes over the duration of a visualization session. Most of the interactive systems fulfil this characteristic. In our method, the user-made selections in image space are linked with all necessary contextual information so that they remain meaningful during the whole session.
